# A major QTL identification and candidate gene analysis of watermelon fruit cracking using QTL-seq and RNA-seq

**DOI:** 10.3389/fpls.2023.1166008

**Published:** 2023-05-15

**Authors:** Yuanfeng Zhan, Wei Hu, Huang He, Xuanmin Dang, Songbi Chen, Zhilong Bie

**Affiliations:** ^1^College of Horticulture and Forestry Sciences, Huazhong Agricultural University, Wuhan, China; ^2^Tropical Crops Genetic Resources Institute, Chinese Academy of Tropical Agricultural Sciences, Haikou, China

**Keywords:** fruit cracking, SLAF-seq, QTL-seq, RNA-seq, DEGs

## Abstract

Fruit cracking decreases the total production and the commercial value of watermelon. The molecular mechanisms of fruit cracking are unknown. In this study, 164 recombinant inbred lines (RILs) of watermelon, derived from the crossing of the WQ1 (cracking-sensitive) and WQ2 (cracking-tolerant) lines, were sequenced using specific length amplified fragment sequencing (SLAF-seq). A high-density genetic linkage map was constructed with 3,335 markers spanning 1,322.74 cM, at an average 0.40 cM across whole-genome flanking markers. The cracking tolerance capacity (CTC), depth of fruit cracking (DFC), rind thickness (RT), and rind hardness (RH) were measured for quantitative trait locus (QTL) analysis. Of the four traits analyzed, one major QTL with high phenotypic variation (41.04%–61.37%) was detected at 76.613–76.919 cM on chromosome 2, which contained 104 annotated genes. Differential gene expression analysis with RNA sequencing (RNA-seq) data between the two parents identified 4,508 differentially expressed genes (DEGs). Comparison of the genes between the QTL region and the DEGs obtained eight coexisting genes. Quantitative real-time PCR (qRT-PCR) analysis revealed that these genes were significant differentially expressed between the two parents. These results provide new insights into the identification of QTLs or genes and marker-assisted breeding in watermelon.

## 1 Introduction

Watermelon (*Citrullus lanatus*) belongs to the cucurbit family (Cucurbitaceae) and is a popular fruit worldwide. It contains nutritional compounds such as sugar, lycopene, citrulline, arginine, and glutathione (Collins et al., 2007). In 2021, China produced 60.9 million tonnes of watermelon, accounting for about 76.3% of crops worldwide, making it one of the top 10 watermelon-producing countries (FAO, 2021). Planting watermelon brings huge benefits to farmers in China. However, the fruit cracking of watermelon during pre- and post-harvest increases the production cost and reduces the economic value of the fruit.

Fruit cracking is a physiological disorder that occurs during fruit growth and development in many crops, such as watermelon, tomato, grape, and apple (Wang et al., 2021b). It is a complex trait associated with morphological, environmental, and genetic factors (Khadivi-Khub, 2015; Capel et al., 2017). Morphological factors, such as shape and rind thickness, affect the stability of the peel (Khadivi-Khub, 2015). Environmental factors, such mineral nutrition, endogenous hormones, water, and temperature, are associated with fruit cracking. The gibberellic acid inhibitor uniconazole P was reported to significantly reduce fruit cracking, whereas the application of excessive nitrogen fertilizer increased fruit cracking (Shimizu, 2005). Water in both the soil and the fruit may influence fruit cracking (Beyer et al., 2002; Gibert et al., 2007). Sudden moisture and temperature changes dramatically increase the fruit temperature and exacerbate fruit cracking (Simon, 2006). Regarding genetic factors, the heritability of fruit cracking in different genetic generations significantly varies (Qi et al., 2015), and different cultivars show different cracking susceptibility even under the same environmental conditions (Khadivi-Khub, 2015), indicating that genetic factors play an important role in fruit cracking.

Fruit cracking is controlled by quantitative trait loci (QTLs) (Vaidyanathan et al., 2006). A large number of QTLs related to fruit cracking have been studied in tomato, sweet cherry, and grape (Capel et al., 2017; Kunihisa et al., 2019; Crump et al., 2022; Zhang et al., 2022). However, for watermelon, only a few QTLs or genes related to fruit cracking have been mapped using bulked segregant analysis (BSA-seq) and QTL mapping (Sun et al., 2020; Yang et al., 2021; Osae et al., 2022). For example, *ClERF4* was shown to be associated with variability in fruit rind hardness (Liao et al., 2020). RNA sequencing (RNA-seq) analysis was used to screen out eight differentially expressed genes (DEGs) between cracking-resistant and cracking-susceptible parents (Jiang et al., 2019). However, the lack of experimental methods to induce cracking has made characterizing this trait a challenge (Capel et al., 2017). Thus, more QTLs related to fruit cracking must be identified.

In this study, the inbred lines WQ1 (cracking-sensitive) and WQ2 (cracking-tolerant) were crossed to generate 164 recombinant inbred lines (RILs). The RILs were then sequenced using specific length amplified fragment sequencing (SLAF-seq) and a high-density genetic map was constructed. Four fruit cracking-related traits—cracking tolerance capacity (CTC), depth of fruit cracking (DFC), rind thickness (RT), and rind hardness (RH)—were evaluated 28 days after pollination (DAP). One major QTL detected for these four traits with high phenotypic variation explained (PVE; 41.04%–61.37%) was found to be located on chromosome 2 and harbored 104 candidate genes. A comparison of the genes in the genetic region and those identified by RNA-seq revealed eight coexisting genes. Quantitative real-time PCR (qRT-PCR) analysis revealed that the eight coexisting genes were significantly differentially expressed between the two parents. Thus, these results provide new insights into mapping or cloning the QTLs or genes of fruit cracking-related traits and could be useful in marker-assisted breeding (MAS).

## 2 Materials and methods

### 2.1 Plant materials and population development

An F_8_ RIL population consisting of 164 lines was generated by self-crossing the watermelon inbred lines WQ1 (female parent, cracking-sensitive) and WQ2 (male parent, cracking-tolerant). WQ1 and WQ2 were from the Tropical Crop Germplasm Research Institute, Chinese Academy of Tropical Agricultural Sciences.

### 2.2 Trait measurement

The F_8_ lines and the two parents were planted in a greenhouse in 2021 (Danzhou, Hainan, China). All plants were grown in wide–narrow rows, with 0.45 m between plants and within a wide row of 1.0 m and a narrow row of 0.5 m. The second or third female flower per plant was artificially pollinated, and the date of pollination was recorded. Only one fruit was reserved for each plant. Field management followed normal watermelon production practices.

The RH, CTC, DFC, and RT of WQ1, WQ2, and the RILs were measured at 28 DAP. Three mature fruits per line were harvested for trait measurement. The mechanical properties of the rinds were measured using TA.XTplus Texture Analyzer (Stable Micro Systems Ltd., Godalming, UK). Three sites on the equatorial zone of each fruit were selected for RH measurement using a P/2E probe. The measurement parameters were set as described (Liao et al., 2020): the pretest speed was 1.00 m/s, the test speed was 2.00 mm/s, the posttest speed was 10 mm/s, and the distance was 15 mm. The RH value was obtained by quantifying the texture characteristic curve. CTC was measured with a knife probe (HDP/BS-B probe). Only one site on the equatorial zone of each fruit (on the reverse side of the RH measuring point) was analyzed. The measurement parameters were the same as those of the RH measurement. The breaking force when the pressure has a sudden decrease was defined as the CTC value. DFC represented the distances (in millimeters) from the contact of the knife probe to the breaking of the rind. The DFC value was calculated as the time multiplied by the test speed. RT was measured with a digital display Vernier caliper as described (Ma and Liu, 2005). For each trait, the average value of three biological replicates was calculated and taken as the phenotypic value.

To evaluate the pericarp morphology of WQ1 and WQ2 growing under normal field conditions, fruits were picked at 10 and 18 DAP. Each genotype contained three biological replicates with two fruits included. The fresh mesocarp was cut out (0.5 cm × 0.5 cm) and fixed with 5% FAA fixative (38% formaldehyde/glacial acetic acid/70% alcohol, 5:5:90, by volume), as described (Guo et al., 2020). The samples were washed twice with 50% ethanol (each time for 10 min) and dehydrated twice through an ascending ethanol series (50%, 70%, 80%, 90%, and 100%, each time for 15 min). Subsequently, the samples were treated with 100% ethanol and xylene (3:1, 1:1, and 1:3, by volume) and 100% xylene, and with xylene and paraffin (3:1, 1:1, and 1:3, by volume) and paraffin (two times, each time at 55°C). Finally, 10-μm-thick paraffin sections were produced. A Zeiss biological microscope was used to observe and photograph the sections. Six fields of view were randomly selected for each sample. ImagePro plus 6.0 software was used to evaluate the structure of the pericarp, including the length and thickness of the epidermal cell, thickness of the exocarp, and areas of the exocarp and mesocarp cells.

### 2.3 DNA extraction

Young leaves from the two parents and 164 RILs were collected for genomic DNA extraction using a modified CTAB method (Saghai-Maroof et al., 1984). DNA was quantified with a NanoDrop 2000 spectrophotometer (NanoDrop, Wilmington, DE, USA) and evaluated by electrophoresis in 1.0% agarose gel. High-quality DNA samples were stored at −20°C until sequencing.

### 2.4 SLAF library construction and high-throughput sequencing of the RILs

The SLAF-seq strategy was used in this study (Biomarker Technologies, Beijing, China) (Sun et al., 2013). Genomic DNA from 164 RILs was digested with *Hae*III and *Hpy*166II [New England Biolabs (NEB), Ipswich, MA, USA]. Subsequently, a single nucleotide (A) was added to the 3′ end of the digested fragments using Klenow Fragment (NEB) and dATP. T4 DNA ligase was used to ligate the Duplex tag-labeled sequencing adapters to distinguish them from raw sequencing data. PCR was performed using forward (5′-AATGATACGGCGACCACCGA-3′) and reverse (5′-CAAGCAGAAGACGGCATACG-3′) primers. The PCR products were purified and pooled. The pooled samples were then separated using 2% agarose gel electrophoresis. Fragments ranging from 314 to 414 bp (with indices and adaptors) were excised and purified using the QIAquick Gel Extraction Kit (Qiagen, Hilden, Germany). Paired-end sequencing (126 bp from both ends) was performed using an Illumina HiSeq 2500 System (Illumina, Inc., San Diego, CA, USA) according to the manufacturer’s instructions. To evaluate the accuracy of the SLAF libraries, the japonica rice Nipponbare (*Oryza sativa* L.) (http://rice.plantbiology.msu.edu/) was used as a control with the same process of library construction and sequencing.

### 2.5 SNP identification and genotyping

SLAF marker identification and genotyping were performed as described (Sun et al., 2013). Low-quality reads with quality scores <20e were filtered out. Clean reads were obtained by trimming the barcodes and terminal 5-bp positions and were then mapped onto the watermelon genome (http://cucurbitgenomics.org/ftp/genome/watermelon/97103/v2/) using BWA software (Li, 2013). Sequences mapping to the same position with >95% identity were defined as one SLAF locus (Zhang et al., 2015). The alleles of each SLAF locus were defined according to parental reads with a sequence depth of greater than fivefold; however, for each offspring, reads with a sequence depth of greater than twofold were used to define alleles. All polymorphic SLAF loci were genotyped with consistency in the parental and offspring SNP loci. Because the RIL populations were constructed using two inbred lines, segregation type aa × bb was used to genotype the SLAF markers in the RILs. The parental genotypes were aa (WQ1) and bb (WQ2), and the offspring genotypes were aa and bb (ab was removed). Polymorphic SLAF markers with the following characteristics were filtered out: 1) parental sequencing depth less than fivefold; 2) number of SNPs >3; 3) integrity filtering to screen markers covered <60% of all offspring genotypes; and 4) missing parental information on filtered sites.

### 2.6 Construction of the high-density genetic map

The polymorphic SLAF markers were assigned to different chromosomes by aligning with the watermelon genome. Each chromosome was considered a linkage group (LG). The modified logarithm of odds (MLOD) scores between markers were calculated to confirm the robustness of the markers for each LG. Markers with MLOD scores <3 were filtered out. The HighMap software (http://highmap.biomarker.com.cn/) was used to order the SLAF markers and correct the genotyping errors within LGs (Liu et al., 2014). Map distances were estimated using the Kosambi mapping function (Kosambi, 2016).

### 2.7 QTL mapping

QTLs were identified using composite interval mapping (CIM) with the R/QTL package (Broman et al., 2003). LOD values were determined based on the 1,000-permutation test. A marker with an LOD value of 3 was considered a putative QTL related to a certain trait in a genomic region.

### 2.8 RNA-seq analysis

For WQ1, a few fruits began to crack at 15 DAP. To identify more DEGs before and after fruit cracking, the rinds of WQ1 and WQ2 fruits that were artificially pollinated at 10 and 18 DAP (before and after fruit cracking, respectively) were collected for RNA extraction. Total RNA was extracted using the TransZol Up Plus RNA Kit, qualified by electrophoresis with the Agilent Bioanalyzer 2100 (Agilent Technologies, Santa Clara, CA, USA), and purified using the RNAClean XP Kit and RNase-Free DNase Set. RNA-seq libraries were constructed after ribosomal RNA (rRNA) removal, fragmentation, first- and second-strand complementary DNA (cDNA) synthesis, end repair, A tailing to the 3′ end, ligation of adapters, and enrichment by PCR amplification. The concentration and the size of the constructed library were detected using Qubit 2.0 Fluorometer and Agilent 4200, respectively. Paired-end (150 bp) sequencing was performed with the Illumina HiSeq X Ten System following the manufacturer’s protocol.

Adaptors and low-quality bases of the raw reads were removed with the FASTX-Toolkit. Short reads of <25 bp were discarded. Clean reads were mapped to the watermelon_97103_v2 genome by HISAT2 (Kim et al., 2015), allowing four mismatches, and unique matches were used to calculate the gene read number and fragments per kilobase of transcript per million fragments mapped (FPKM) (Trapnell et al., 2010).

### 2.9 Differentially expressed genes

Two groups (WQ1 *vs*. WQ2 at 10 DAP and WQ1 *vs*. WQ2 at 18 DAP) were used to detect the DEGs. DEGs were screened out using the DESeq2 package in R (Love et al., 2014). A fold change ≥2 and a *q*-value ≤0.05 were set as the cutoff values.

### 2.10 Expression analysis of the candidate genes

Total RNA was extracted from the rind of WQ1 and WQ2 at 10 DAP using the TRIzol reagent (Invitrogen, Carlsbad, CA, USA). Thereafter, cDNA was synthesized from 3 μg gDNA Eraser-treated (Takara, San Jose, CA, USA) RNA. cDNA corresponding to 30 ng of the total RNA was used as the template for each SYBR Green PCR reaction, which utilized the ABI ViiA 7 Real-Time PCR System (Applied Biosystems, Foster City, CA, USA). qRT-PCR was performed with three biological and three technical replicates for each candidate gene. *Cla97C05G094190* (*GAPDH*) was used as the internal control (Itoh et al., 2023). The primers for qRT-PCR were designed using Primer 5 (Lalitha, 2000) and are listed in **Supplementary Table S1**.

## 3 Results

### 3.1 Phenotypic analysis of the parents and RILs

**Figure 1:**
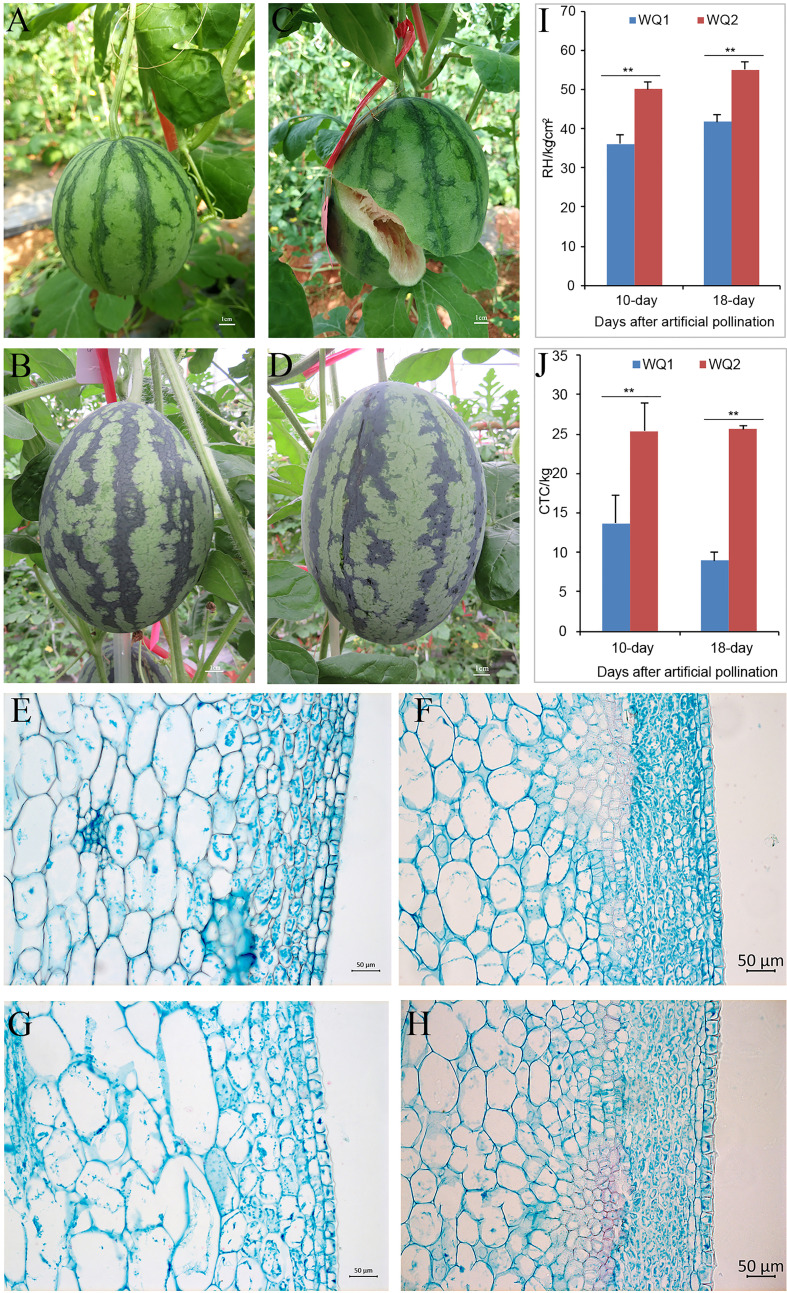
Phenotypes of parents. **(A, B)** WQ1 and WQ2 at 10 days after pollination (DAP). **(C, D)** WQ1 and WQ2 at 18 DAP. **(E, F)** Pericarp morphology of WQ1 and WQ2 at 10 DAP. **(G, H)** Pericarp morphology of WQ1 and WQ2 at 18 DAP. **(I, J)** Rind hardness (RH) and cracking tolerance capacity (CTC) of fruits at 10 and 18 DAP. **p < 0.05.

WQ1 is a fruit cracking-sensitive inbred line that has a rate of 77.8% fruit cracking in the field (**Figures 1A, C**), while WQ2 is an inbred line that is fruit cracking-tolerant (**Figures 1B, D**). The RH and CTC values of WQ2 at 10 and 18 DAP were significantly higher than those of WQ1 (**Figures 1I, J**). The CTC values of WQ2 were 1.85 and 2.85 times higher than those of WQ1 at 10 and 18 DAP, respectively (**Figures 1I, J**). The RH values of both WQ1 and WQ2 increased after artificial pollination. The CTC value of WQ1 decreased at 18 DAP, whereas that of WQ2 increased.

The length of the epidermal cells in WQ1 was significantly higher than that in WQ2 at each stage (at 10 and 18 DAP), but the thickness of the epidermal cells was only significantly different between WQ1 and WQ2 at 18 DAP. The epidermal cells of WQ2 were almost square, and their size showed little change during fruit development. The length-to-thickness ratio varied from 1.10 to 1.21. In contrast, the epidermal cells of WQ1 were rectangular, and the cells were elongated with fruit development. The length-to-thickness ratio changed from 1.57 to 2.11 (**Figures 1E–H** and **Supplementary Table S2**).

The exocarp of the fruit in WQ2 had 9–12 layers of cells, and its thickness changed from 119.01 to 137.69 μm at a different stage. The exocarp cells were small and closely arranged and short oval or short polygonal in shape. In contrast, the exocarp of the fruit in WQ1 was thinner and consisted of four to six layers of cells, with its thickness changing from 76.29 to 78.17 μm at a different stage. The exocarp cell area of WQ1 cells was 1.75–3.18 times larger than that of WQ2 cells (**Figures 1E–H** and **Supplementary Table S2**).

The mesocarp cell area of WQ1 was significantly larger than that of WQ2. The mesocarp cells of WQ2 and WQ1 gradually became larger with the development of the fruit. However, the mesocarp cells of WQ2 changed little during fruit development, with the cell area increasing by only 133.79 μm^2^ from 10 to 18 DAP. However, the mesocarp cells of WQ1 changed greatly during fruit development. The area was 2,238.93 μm^2^ at 10 DAP, which increased to 3,925 μm^2^ at 18 DAP (**Figures 1E–H** and **Supplementary Table S2**).

**Figure 2:**
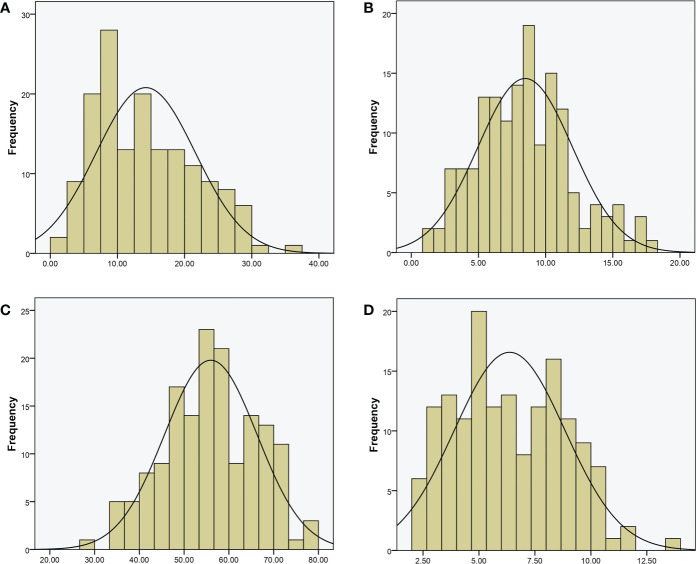
Phenotypic distribution of recombinant inbred lines (RILs). **(A)** Cracking tolerance capacity (CTC, in kilograms). **(B)** Depth of fruit cracking (DFC, in millimeters). **(C)** Rind hardness (RH in kilograms per square centimeter); **(D)** rind thickness (RT in millimeters).

For QTL mapping, the CTC, DFC, RT, and RH of the RILs and WQ1 and WQ2 were measured at 28 DAP. Significant differences were observed in the fruit cracking-related traits between the two parents and the RILs (**Table 1**). The average CTC, DFC, RH, and RT values of the RILs were 14.23 kg (1.58–37.20 kg), 8.48 mm (1.51–18.17 mm), 55.95 kg (29.40–78.45 kg), and 6.37 mm (2.09–13.74 mm), respectively. All four traits exhibited super-parent segregation and normal distribution in the RILs (**Figure 2**). The correlation between the four traits showed significant differences (**Supplementary Table S3**), indicating that these traits were closely related to fruit cracking.

**Table 1:** Fruit-cracking traits related to the parents and RILs.

Trait	Parents		RILs		VC (%)	Skewness	Kurtosis
	WQ1	WQ2	Average	Range			
CTC (kg)	4.52	24.64**	14.23	1.58–37.20	52.07	0.579	−0.421
DFC (mm)	3.11	14.81**	8.48	1.51–18.17	41.58	0.399	−0.050
RH (kg/cm^2^)	45.92	60.58**	55.95	29.40–78.45	18.65	−0.107	−0.549
RT (mm)	3.18	8.62**	6.37	2.09–13.74	39.08	0.284	−0.665

Asterisks mark significant differences according to Student’s t-test.

CTC, cracking tolerance capacity; DFC, depth of fruit cracking; RH, rind hardness; RIL, recombinant inbred line; RT, rind thickness; VC, variable coefficient.

**p < 0.01.

### 3.2 Construction of the genetic map

After the clean reads were mapped to the watermelon reference genome, 197,639 and 198,988 SNP markers were obtained from WQ1 and WQ2, respectively. A total of 494,293 SNP markers were developed between the parents and the RILs (**Supplementary Table S4**). Finally, 175,062 SNP markers were obtained by filtering out those markers that had no polymorphism between the parents or the SNPs that were missing in the offspring or distributing partial separation (**Supplementary Table S5**). The filtered SNPs were classified into seven types (i.e., aa × bb, ab × cc, cc × ab, ef × eg, hk × hk, lm × ll, and nn × np) (**Supplementary Figure S1**). Because the population used in this study was the RIL population constructed by two diploid inbred lines, the aa × bb type (132,967 SNP markers) was reserved for genetic analysis. After fine filtering, 4,857 SNP markers were finally obtained for the construction of the genetic map.

**Figure 3:**
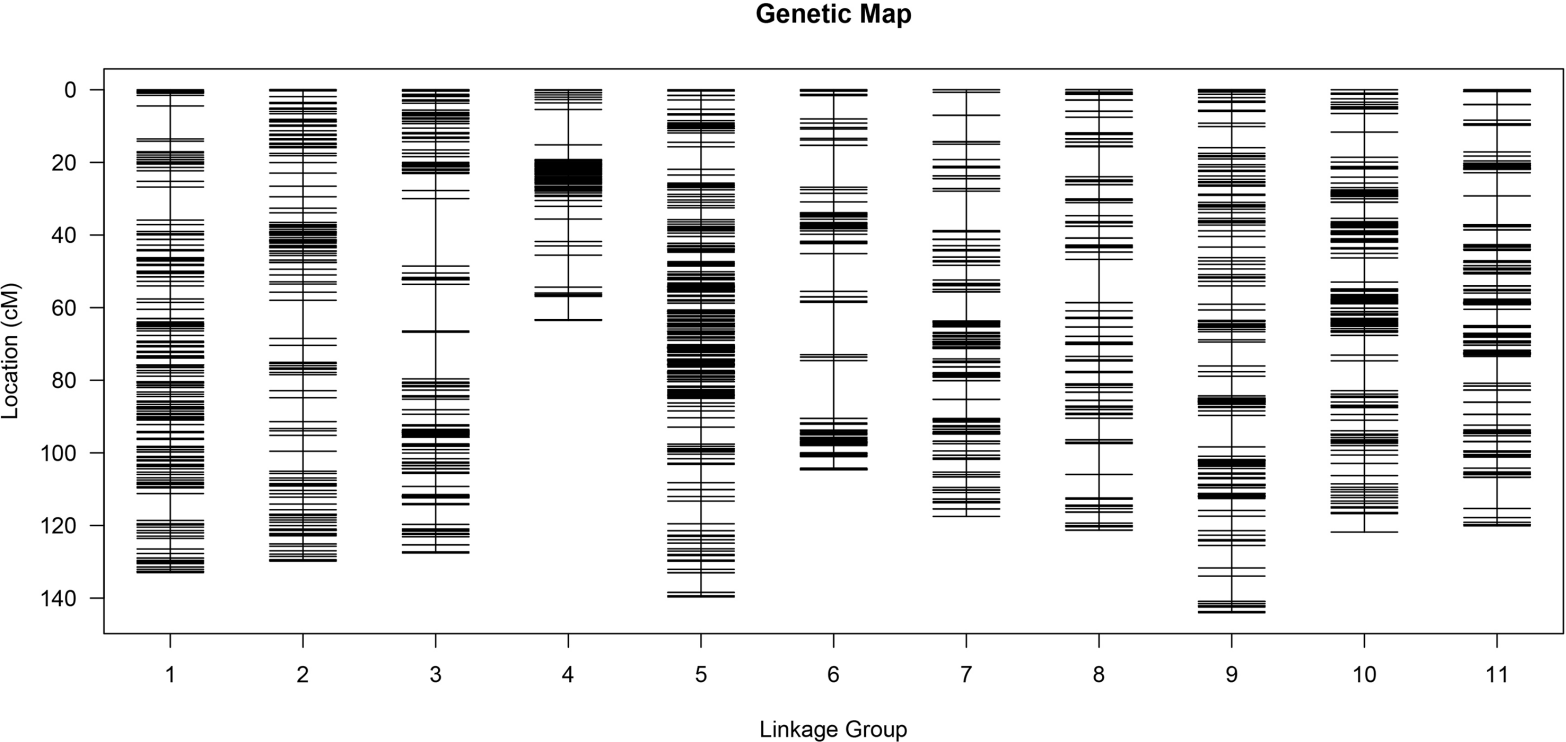
High-density genetic map.

The MLOD values between the two markers were calculated for the 4,857 SNP markers. The highest MLOD values between the markers were classified into the same LG. A total of 3,335 SNP markers were obtained, accounting for 68.66% of the total markers. A high-density genetic map including 11 chromosomes was constructed using HighMap software (**Figure 3**). The number of SNP markers in each chromosome ranged from 113 to 538. A genetic map with 1,322.74 cM length was developed, ranging from 63.48 to 144.02 cM among all the chromosomes. The average genetic distance between the SNP markers across the chromosomes was 0.4 cM. The average genetic distances on chromosomes 2 (0.73 cM) and 5 (0.26 cM) were the largest and smallest across all the chromosomes, respectively. The ratio of gaps ≤5 cM in each chromosome ranged from 96.43% to 99.26% (**Supplementary Table S6**). Generally, the higher the ratio of gaps <5 cM to the total number of gaps, the more uniform the map. The maximum distance on different chromosomes ranged from 6.24 to 18.58, of which the maximum distance between chromosome 5 was the smallest and that of chromosome 3 was the largest (**Supplementary Table S7**).

### 3.3 Detection of QTLs associated with fruit cracking

**Figure 4:**
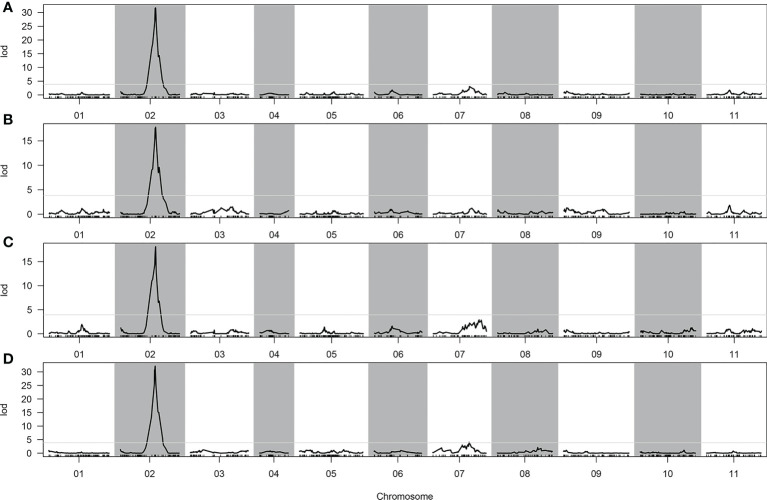
Quantitative trait loci (QTLs) detected in the whole genome. **(A)** Cracking tolerance capacity (CTC). **(B)** Depth of fruit cracking (DFC). **(C)** Rind hardness (RH). **(D)** Rind thickness (RT).

In total, two, one, one, and two QTLs were detected for CTC, DFC, RT, and RH, respectively, when the LOD threshold was set to 3 (**Table 2**). Of all the detected QTLs, one locus with high PVE (41.04%–61.37%) was detected for each of the four traits, which was located at 76.613–76.919 cM on chromosome 2 (**Figure 4**). The physical location at this interval was 31.804–32.805 Mb. A total of 104 genes were predicted in this interval. All the additive effects of this QTL were negative, indicating that the WQ2 allele of this locus is the desirable one for fruit cracking resistance. Another minor QTL, which just exceeded the LOD thresholds for CTC and RT, was detected on chromosome 7 at the same position, with PVE of 8.66% and 11.2% (**Table 2**).

**Table 2:** QTLs detected of the CTC, DFC, RT, and RH.

QTL	Max LOD score	Group	Genetic position (cM)	Additive	PVE (%)
*qCTC-1*	31.7	2	76.613–76.919	−5.765	60.83
*qCTC-2*	3.1	7	80.127	2.174	8.66
*qDFC*	17.8	2	76.613–76.919	−2.253	41.04
*qRH*	18.1	2	76.613–76.919	−6.677	41.6
*qRT-1*	32.1	2	76.613–76.919	−1.937	61.37
*qRT-2*	4	7	77.907–80.127	0.829	11.2

CTC, cracking tolerance capacity; DFC, depth of fruit cracking; LOD, limit of detection; PVE, phenotypic variation explained; QTL, quantitative trait locus; RH, rind hardness; RIL, recombinant inbred line; RT, rind thickness.

### 3.4 RNA-seq analysis

For each repeat, >57,616,037 clean reads were obtained, with matching rates ranging from 80.17% to 86.04%. The proportion of reads matching multiple positions was between 1.05% and 1.53% (<10%), indicating that the sequencing results of all samples were of high quality for subsequent analysis (**Supplementary Table S7**).

In total, 2,551 DEGs were detected between WQ1 and WQ2 at 10 DAP, with 1,342 upregulated and 1,209 downregulated DEGs (**Supplementary Figure S2A** and **Supplementary Data Sheet 1**). In addition, 3,642 DEGs were detected between WQ1 and WQ2 at 18 DAP, with 1,963 DEGs that were upregulated and 1,679 that were downregulated (**Supplementary Figure 1B** and **Supplementary Data Sheet 2**). In the DEGs detected in the two groups, 1,685 genes were co-detected. A total of 866 and 1,957 unique DEGs were detected at 10 and 18 DAP, respectively (**Supplementary Figure S3**).

### 3.5 Gene ontology analysis of DEGs

**Figure 5:**
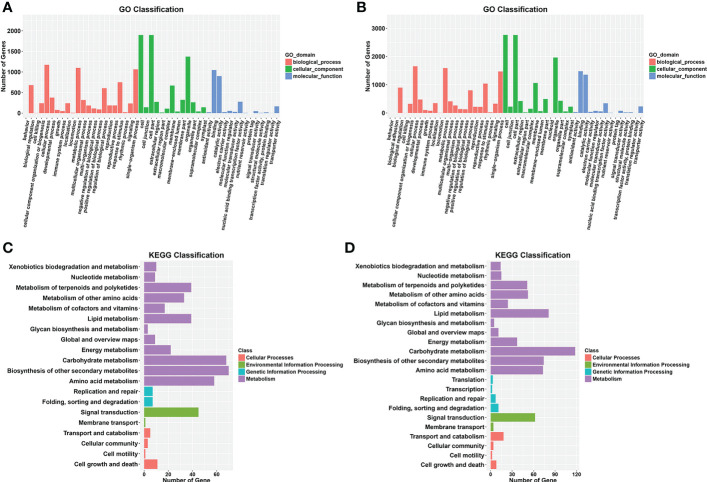
Gene Ontology (GO) and Kyoto Encyclopedia of Genes and Genomes (KEGG) enrichment analyses of the differentially expressed genes (DEGs) from the two comparison groups. **(A, B)** GO enrichment analysis between WQ1 and WQ2 at 10 and 18 days after pollination (DAP). **(C, D)** KEGG enrichment analysis between WQ1 and WQ2 10 at and 18 DAP.

Gene Ontology (GO) analysis was conducted for the functional classification of the DEGs. In total, 2,290 (89.7%) DEGs were assigned to three GO classes—biological process, cellular component, and molecular function—with 2,289 DEGs classified into 49 functional groups in the WQ1 *vs*. WQ2 at 10 DAP group (**Figure 5A**). In biological process, the DEGs were mainly classified into cellular process (*n* = 1,175), material metabolism (*n* = 1,100), and single-organism process (*n* = 1,064). In cellular component, the DEGs were mainly classified into cells (*n* = 1,898), cell parts (*n* = 1,898), and organelles (*n* = 1,373). In molecular function, the DEGs were mainly classified into protein binding (*n* = 1,047) and enzyme catalysis activity (*n* = 899).

For the WQ1 *vs*. WQ2 at 18 DAP group, 3,230 (88.7%) DEGs were assigned to three GO classes, with 2,290 DEGs classified into 51 functional groups (**Figure 5B**). In biological process, the DEGs were mainly classified into cellular process (*n* = 1,655), material metabolism (*n* = 1,588), and single-organism process (*n* = 1,471). In cellular component, the DEGs were mainly classified into cells (*n* = 2,764), cell parts (*n* = 2,762), and organelles (*n* = 1,964). In molecular function, the DEGs were mainly classified into protein binding (*n* = 1,480) and enzyme catalysis activity (*n* = 1,355).

### 3.6 KEGG analysis of DEGs

The Kyoto Encyclopedia of Genes and Genomes (KEGG) categories involved cellular processes, environmental information processing, genetic information processing, metabolism, and organismal systems. A total of 309 DEGs were assigned to 92 KEGG pathways in the WQ1 *vs*. WQ2 at 10 DAP group (**Supplementary Data Sheet 3**). The significantly enriched pathways included phenylpropanoid biosynthesis; flavonoid biosynthesis; secondary metabolite biosynthesis; phenylalanine metabolism; and biosynthesis of stilbene, heptane, and gingerol (**Figure 5C**).

In total, 450 DEGs were assigned to 107 KEGG pathways in the WQ1 *vs*. WQ2 at 18 DAP group (**Supplementary Data Sheet 4**). The significantly enriched pathways included pentose and gluconate interconversion; phenylalanine biosynthesis; cutin, flavin, and wax biosynthesis; and linoleic acid metabolism (**Figure 5D**).

### 3.7 Selection of candidate genes

**Figure 6:**
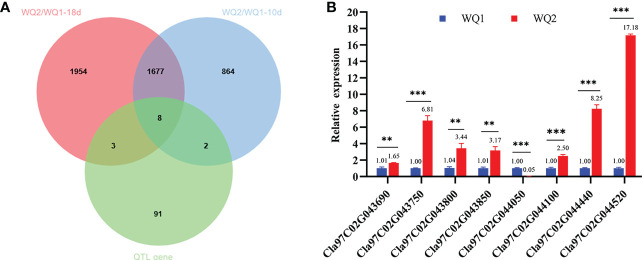
Coexisting genes in quantitative trait locus sequencing (QTL-seq) and RNA-seq and the candidate genes identified by quantitative real-time PCR (qRT-PCR). **(A)** Venn diagram of the differentially expressed genes at 10 and 18 days after pollination (DAP) and the genes within the QTL intervals. **(B)** Relative expression of the candidate genes between the two parents. *M*, maternal (WQ1); *P*, paternal (WQ2). **p < 0.01, **p < 0.001.

The comparison of the DEGs detected through RNA-seq and the genes on the major QTL interval obtained eight coexisting genes, of which seven were upregulated and one was downregulated (**Table 3**). The eight coexisting genes included four transcription factors (*Cla97C02G043800*, *Cla97C02G044050*, *Cla97C02G044440*, and *Cla97C02G044520*) and four other annotated genes (*Cla97C02G043690*, *Cla97C02G043750*, *Cla97C02G043850*, and *Cla97C02G044100*) (**Figure 6A** and **Table 3**). The coexisting genes were verified by qRT-PCR. Among the seven upregulated genes, the expression of *Cla97C02G044520* and *Cla97C02G043690* between WQ1 and WQ2 showed the largest and the smallest difference, respectively. For the only downregulated gene, *Cla97C02G044050*, the expression of WQ1 was 20 times of WQ2 (**Figure 6B**). The expression of all detected genes reached significant levels.

**Table 3:** Coexisting genes detected by comparing the QTL-seq and RNA-seq.

Gene ID	Description	P/S difference
*Cla97C02G043690*	Reticulon-like protein	UP
*Cla97C02G043750*	Seed biotin-containing protein SBP65	UP
*Cla97C02G043800*	Transcription factor TCP4-like	UP
*Cla97C02G043850*	Nucleobase-ascorbate transporter 1	UP
*Cla97C02G044050*	Heat stress transcription factor A-4c-like	DOWN
*Cla97C02G044100*	Serine/threonine protein phosphatase 7 long form	UP
*Cla97C02G044440*	Zinc finger protein CONSTANS	UP
*Cla97C02G044520*	MYB transcription factor 58.1	UP

UP, upregulated; DOWN, downregulated.

## 4 Discussion

Flesh fruits, such as apple, sweet cherry, grape, and tomato, can crack or split during growth and development, causing severe economic loss (Khadivi-Khub, 2015). QTLs related to fruit cracking have been mapped for some fruits with different populations (Capel et al., 2017; Kunihisa et al., 2019; Crump et al., 2022; Zhang et al., 2022). However, only a few studies have focused on the fruit cracking of watermelon (Jiang et al., 2019; Liao et al., 2020; Sun et al., 2020).

Accurate measurement of phenotypes is important for QTL mapping. Only a few QTLs have been identified by counting the number of cracking fruits, calculating the cracked fruit rate, or evaluating the melon cracking capacity with high levels of irrigation (Huang et al., 1999; Qi et al., 2015; Capel et al., 2017). However, no QTL with a large effect was identified at the same time, which is unfavorable for gene identification or applications. RH has been reported as a reliable indicator of cracking resistance capacity (Liao et al., 2020). The CTC, DFC, RT, and RH values showed high correlations with each other in the correlation analysis (**Supplementary Table S3**). One major locus was mapped to the same region by quantitative trait locus sequencing (QTL-seq), indicating that, in addition to RH, the phenotype of CTC, DFC, and RT can be used for the QTL mapping of fruit cracking.

Compared with traditional molecular markers—such as random amplified polymorphic DNA (RAPD), restriction fragment length polymorphism (RFLP), cleaved amplified polymorphic sequence (CAPS), or simple sequence repeat (SSR) markers—a lot of markers can be detected through high-throughput sequencing. In this study, a genetic map of 1,322.74 cM length was constructed, which had a higher density than the map developed using molecular markers (Hashizume et al., 2003; Cheng et al., 2016; Yang et al., 2021; Osae et al., 2022). However, the maximum gap on chromosomes 3 and 6 was relatively large (**Supplementary Table S6**), indicating that the sequencing fold may have been increased. High-density genetic maps in watermelons have been rarely reported.

QTL-seq and RNA-seq have been used for QTL identification in many plants (Park et al., 2019; Wen et al., 2019; Lei et al., 2020; Xue et al., 2022). However, for some agronomic traits, it is almost impossible to identify the QTLs using RIL or F_2_ populations due to fewer recombination sites and complex genetic backgrounds. Although a minor QTL was identified on chromosome 7, it had little contribution to the fruit cracking capacity between the two parents (**Table 2**). Therefore, the QTL located on chromosome 2 can be considered a quality trait locus, which can be fine-mapped using F_2:3_ populations or introgression lines.

During fruit development, the pericarp structures of WQ2 and WQ1 were significantly different (**Figures 1E–H**). The RH and CTC values of WQ2 were consistently significantly higher than those of WQ1. The thickness of the pericarp and exocarp of WQ2 was significantly higher than that of WQ1, and the lengths and areas of the epicarp and mesocarp cells of WQ1 were significantly greater than those of WQ2 (**Supplementary Table S2**). In later fruit development, the length of the epidermal cells, the area of the epicarp cells, and the area of the mesocarp cells of WQ1 were 2.91, 3.28, and 5.69 times larger than those of WQ2, respectively. The RH and CTC values were significantly positively correlated with pericarp and exocarp thickness, but significantly negatively correlated with epidermal cell length and the exocarp and mesocarp cell areas. Therefore, RH and CTC can be used to measure the cracking resistance of watermelon fruit, which is more appropriate at the later stage of fruit development.

In total, 866 and 1,957 unique DEGs were obtained at 10 and 18 DAP, respectively (**Figure 6A**), indicating that, with fruit development, the gene expression was changed. The changed DEGs may be responsible for fruit cracking. DEGs changing during fruit development may play a key role in the difference between watermelon rind and fruit cracking. GO and KEGG enrichment analyses of the DEGs before and after cracking (at 10 and 18 DAP) revealed that lignin catabolism, glucuronoxylan synthesis and metabolism, phenylpropanoid biosynthesis, flavonoid biosynthesis, secondary metabolite biosynthesis, plant-type secondary cell wall biogenesis, and regulation of jasmonic acid-mediated signaling pathways were significantly enriched. The change of DEGs may play a vital role in the formation of the rind tissue structure of watermelon during fruit development. Of the eight coexisting candidate genes both in the QTL interval and DEGs, four were transcription factors. Transcription factors are involved in the regulation of floral and fruit development and gametophyte cell division (Lai et al., 2020). Many transcription factors have been detected in fruit cracking through RNA-seq (Jiang et al., 2019; Wang et al., 2021a). This may provide new insights into the selection of candidate genes.

In future studies, our group aims to construct F_2:3_ populations or introgression lines for the fine-mapping of major QTLs. In addition, genetic transformation of the candidate genes will be performed. The results of this study may provide a useful locus for MAS and gene function studies.

## Data availability statement

The datasets presented in this study can be found in online repositories. The names of the repository/repositories and accession number(s) can be found below: https://www.ncbi.nlm.nih.gov/genbank/, SRR23013875–SRR23014040.

## Author contributions

YZ, SC, and ZB conceived the research. YZ, WH, HH, and XD performed the experiments and analyzed the data. WH and YZ drafted the manuscript. XD, SC, and ZB revised the manuscript. All authors contributed to the article and approved the submitted version.

## References

[B1] Beyer M. Peschel S. Knoche M. Knoürgen M. (2002). Studies on water transport through the sweet cherry fruit surface: IV. regions of preferential uptake. HortScience 37 (4), 637–641. doi: 10.21273/HORTSCI.37.4.637

[B2] Broman K. W. Wu H. Sen Ś Churchill G. A. (2003). R/qtl: QTL mapping in experimental crosses. Bioinformatics 19 (7), 889–890. doi: 10.1093/bioinformatics/btg112 12724300

[B3] Capel C. Yuste-Lisbona F. J. López-Casado G. Angosto T. Cuartero J. Lozano R.. (2017). Multi-environment QTL mapping reveals genetic architecture of fruit cracking in a tomato RIL solanum lycopersicum× s. pimpinellifolium population. Theor. Appl. Genet. 130, 213–222. doi: 10.1007/s00122-016-2809-9 27742924

[B4] Cheng Y. Luan F. Wang X. Gao P. Zhu Z. Liu S.. (2016). Construction of a genetic linkage map of watermelon (Citrullus lanatus) using CAPS and SSR markers and QTL analysis for fruit quality traits. Sci. Hortic. 202, 25–31. doi: 10.1016/j.scienta.2016.01.004

[B5] Collins J. K. Wu G. Perkins-Veazie P. Spears K. Claypool P. L. Baker R. A.. (2007). Watermelon consumption increases plasma arginine concentrations in adults. Nutrition 23 (3), 261–266. doi: 10.1016/j.nut.2007.01.005 17352962

[B6] Crump W. W. Peace C. Zhang Z. McCord P. (2022). Detection of breeding-relevant fruit cracking and fruit firmness quantitative trait loci in sweet cherry *via* pedigree-based and genome-wide association approaches. Front. Plant Sci. 13. doi: 10.3389/fpls.2022.823250/full PMC8924583 35310633

[B7] FAO (2021). Top 10 country production of watermelon. Available at: https://www.fao.org/faostat/en/#rankings/countries_by_commodity.

[B8] Gibert C. Chadœuf J. Vercambre G. Génard M. Lescourret F. (2007). Cuticular cracking on nectarine fruit surface: spatial distribution and development in relation to irrigation and thinning. J. Am. Soc. Hortic. Sci. 132 (5), 583–591. doi: 10.21273/JASHS.132.5.583

[B9] Guo Y. Zhang T. Zhong J. Ba T. Xu T. Zhang Q.. (2020). Identification of the volatile compounds and observation of the glandular trichomes in opisthopappus taihangensis and four species of chrysanthemum. Plants 9 (7), 855. doi: 10.3390/plants9070855 32640748 PMC7412243

[B10] Hashizume T. Shimamoto I. Hirai M. (2003). Construction of a linkage map and QTL analysis of horticultural traits for watermelon [Citrullus lanatus (THUNB.) MATSUM & NAKAI] using RAPD, RFLP and ISSR markers. Theor. Appl. Genet. 106, 779–785. doi: 10.1007/s00122-002-1030-1 12647050

[B11] Huang X. Wang H. Gao F. Huang H. (1999). A comparative study of the pericarp of litchi cultivars susceptible and resistant to fruit cracking. J. Hortic. Sci. Biotechnol. 74 (3), 351–354. doi: 10.1080/14620316.1999.11511120

[B12] Itoh T. Muramatsu M. Miyazono D. Koketsu M. Fujita S. Hashizume T. (2023). Phenolic glycosides citrulluside h and citrulluside T isolated from young watermelon (Citrullus lanatus) fruit have beneficial effects against cutibacterium acnes-induced skin inflammation. Nat. Prod. Commun. 18 (1), 1934578X221143202. doi: 10.1177/1934578X221143202

[B13] Jiang H. Tian H. Yan C. Jia L. Wang Y. Wang M.. (2019). RNA-Seq analysis of watermelon (Citrullus lanatus) to identify genes involved in fruit cracking. Sci. Hortic. 248, 248–255. doi: 10.1016/j.scienta.2019.01.005

[B14] Khadivi-Khub A. (2015). Physiological and genetic factors influencing fruit cracking. Acta Physiol. Plant 37 (1), 1718. doi: 10.1007/s11738-014-1718-2

[B15] Kim D. Langmead B. Salzberg S. L. (2015). HISAT: a fast spliced aligner with low memory requirements. Nat. Meth. 12 (4), 357–360. doi: 10.1038/nmeth.3317 PMC4655817 25751142

[B16] Kosambi D. D. (2016). “The estimation of map distances from recombination values,” in DD kosambi (New Delhi, India: Springer), 125–130. doi: 10.1007/978-81-322-3676-4_16

[B17] Kunihisa M. Takita Y. Yamaguchi N. Okada H. Sato M. Komori S.. (2019). The use of a fertile doubled haploid apple line for QTL analysis of fruit traits. Breed. Sci. 69 (3), 410–419. doi: 10.1270/jsbbs.18197 31598073 PMC6776154

[B18] Lai X. Chahtane H. Martin-Arevalillo R. Zubieta C. Parcy F. (2020). Contrasted evolutionary trajectories of plant transcription factors. Curr. Opin. Plant Biol. 54, 101–107. doi: 10.1016/j.pbi.2020.03.002 32417720

[B19] Lalitha S. (2000). Primer premier 5. Biotech. Software. Internet Report.: Comput. Software. J. Sci. 1 (6), 270–272. doi: 10.1089/152791600459894

[B20] Lei L. Zheng H. Bi Y. Yang L. Liu H. Wang J.. (2020). Identification of a major QTL and candidate gene analysis of salt tolerance at the bud burst stage in rice (Oryza sativa l.) using QTL-seq and RNA-seq. Rice 13, 1–14. doi: 10.1186/s12284-020-00416-1 32778977 PMC7417472

[B21] Li H. (2013). Aligning sequence reads, clone sequences and assembly contigs with BWA-MEM. arXiv. preprint. arXiv:1303.3997. doi: 10.48550/arXiv.1303.3997

[B22] Liao N. Hu Z. Li Y. Hao J. Chen S. Xue Q.. (2020). Ethylene-responsive factor 4 is associated with the desirable rind hardness trait conferring cracking resistance in fresh fruits of watermelon. Plant Biotechnol. J. 18 (4), 1066–1077. doi: 10.1111/pbi.13276 31610078 PMC7061880

[B23] Liu D. Ma C. Hong W. Huang L. Liu M. Liu H.. (2014). Construction and analysis of high-density linkage map using high-throughput sequencing data. PloS One 9 (6), e98855. doi: 10.1371/journal.pone.0098855 24905985 PMC4048240

[B24] Love M. I. Huber W. Anders S. (2014). Moderated estimation of fold change and dispersion for RNA-seq data with DESeq2. Genome Biol. 15 (12), 1–21. doi: 10.1186/s13059-014-0550-8 PMC4302049 25516281

[B25] Ma S. Liu J. (2005). Watermelon germplasm resources description specification and data standard (Beijing: China Agricultural Press).

[B26] Osae B. A. Amanullah S. Liu H. Liu S. Saroj A. Zhang C.. (2022). CAPS marker-base genetic linkage mapping and QTL analysis for watermelon ovary, fruit and seed-related traits. Euphytica 218 (4), 39. doi: 10.1007/s10681-022-02990-5

[B27] Park M. Lee J.-H. Han K. Jang S. Han J. Lim J.-H.. (2019). A major QTL and candidate genes for capsaicinoid biosynthesis in the pericarp of capsicum chinense revealed using QTL-seq and RNA-seq. Theor. Appl. Genet. 132, 515–529. doi: 10.1007/s00122-018-3238-8 30426173

[B28] Qi Z. Li J. Raza M. A. Zou X. Cao L. Rao L.. (2015). Inheritance of fruit cracking resistance of melon (Cucumis melo l.) fitting e-0 genetic model using major gene plus polygene inheritance analysis. Sci. Hortic. 189, 168–174. doi: 10.1016/j.scienta.2015.04.004

[B29] Saghai-Maroof M. A. Soliman K. M. Jorgensen R. A. Allard R. (1984). Ribosomal DNA spacer-length polymorphisms in barley: mendelian inheritance, chromosomal location, and population dynamics. Proc. Natl. Acad. Sci. U.S.A. 81 (24), 8014–8018. doi: 10.1073/pnas.81.24.8014 6096873 PMC392284

[B30] Shimizu T. (2005). Effects of GA inhibitor on the fruit cracking of melon (Cucumis melo l.). Hortic. Res. 4, 89–93. doi: 10.2503/hrj.4.89

[B31] Simon G. (2006). Review on rain induced fruit cracking of sweet cherries (Prunus avium l.), its causes and the possibilities of prevention. Int. J. Hortic. Sci. 12 (3), 27–35. doi: 10.31421/IJHS/12/3/654

[B32] Sun X. Liu D. Zhang X. Li W. Liu H. Hong W.. (2013). SLAF-seq: an efficient method of large-scale *de novo* SNP discovery and genotyping using high-throughput sequencing. PloS One 8 (3), e58700. doi: 10.1371/journal.pone.0058700 23527008 PMC3602454

[B33] Sun L. Zhang Y. Cui H. Zhang L. Sha T. Wang C.. (2020). Linkage mapping and comparative transcriptome analysis of firmness in watermelon (Citrullus lanatus). Front. Plant Sci. 11. doi: 10.3389/fpls.2020.00831 PMC7308538 32612625

[B34] Trapnell C. Williams B. A. Pertea G. Mortazavi A. Kwan G. Van Baren M. J.. (2010). Transcript assembly and quantification by RNA-seq reveals unannotated transcripts and isoform switching during cell differentiation. Nat. Biotechnol. 28 (5), 511–515. doi: 10.1038/nbt.1621 20436464 PMC3146043

[B35] Vaidyanathan S. Harrigan G. G. Goodacre R. (2006). Metabolome analyses:: strategies for systems biology (New York, United States: Springer Science & Business Media).

[B36] Wang Y. Guo L. Zhao X. Zhao Y. Hao Z. Luo H.. (2021b). Advances in mechanisms and omics pertaining to fruit cracking in horticultural plants. Agronomy 11 (6), 1045. doi: 10.3390/agronomy11061045

[B37] Wang J. Wu X. Tang Y. Li J. G. Zhao M. (2021a). RNA-Seq provides new insights into the molecular events involved in “Ball-skin versus bladder effect” on fruit cracking in litchi. Int. J. Mol. Sci. 22 (1), 454. doi: 10.3390/ijms22010454 33466443 PMC7796454

[B38] Wen J. Jiang F. Weng Y. Sun M. Shi X. Zhou Y.. (2019). Identification of heat-tolerance QTLs and high-temperature stress-responsive genes through conventional QTL mapping, QTL-seq and RNA-seq in tomato. BMC Plant Biol. 19, 1–17. doi: 10.1186/s12870-019-2008-3 31510927 PMC6739936

[B39] Xue Y. Gao H. Liu X. Tang X. Cao D. Luan X.. (2022). QTL mapping of palmitic acid content using specific-locus amplified fragment sequencing (SLAF-seq) genotyping in soybeans (Glycine max l.). Int. J. Mol. Sci. 23 (19), 11273. doi: 10.3390/ijms231911273 36232577 PMC9569734

[B40] Yang T. Amanullah S. Pan J. Chen G. Liu S. Ma S.. (2021). Identification of putative genetic regions for watermelon rind hardness and related traits by BSA-seq and QTL mapping. Euphytica 217, 1–18. doi: 10.1007/s10681-020-02758-9

[B41] Zhang C. Cui L. Liu C. Fan X. Fang J. (2022). Mining candidate genes of grape berry cracking based on high density genetic map. Hortic. Plant J. doi: 10.1016/j.hpj.2022.10.004

[B42] Zhang J. Zhang Q. Cheng T. Yang W. Pan H. Zhong J.. (2015). High-density genetic map construction and identification of a locus controlling weeping trait in an ornamental woody plant (Prunus mume sieb. et zucc). DNA Res. 22 (3), 183–191. doi: 10.1093/dnares/dsv003 25776277 PMC4463843

