# Supplementary material for: A major QTL identification and candidate gene analysis of watermelon fruit cracking using QTL-seq and RNA-seq
Source: Front Plant Sci. 2023 May 15;14:1166008. doi: 10.3389/fpls.2023.1166008 (PMC10225605; doi:10.3389/fpls.2023.1166008)
Supplement: Supplementary file 1 [file DataSheet_1.docx]

Supplementary Material

A Major QTL Identification and Candidate Gene Analysis of Watermelon Fruit Cracking Using QTL-Seq and RNA-Seq

Yuanfeng Zhan^1,2†^, Wei Hu^2†^, Huang He^2^, Xuanmin Dang^2*^, Songbi Chen^2*^, Zhilong Bie^1*^

*** Correspondence:** Xuanmin Dang ([evergreen088@163.com](mailto:evergreen088@163.com)), Songbi Chen ([songbichen@catas.cn](mailto:songbichen@catas.cn)) and Zhilong Bie ([biezl@mail.hzau.edu.cn](mailto:biezl@mail.hzau.edu.cn))

**Supplementary Figure 1.** Distribution of 7 SNP types.

**
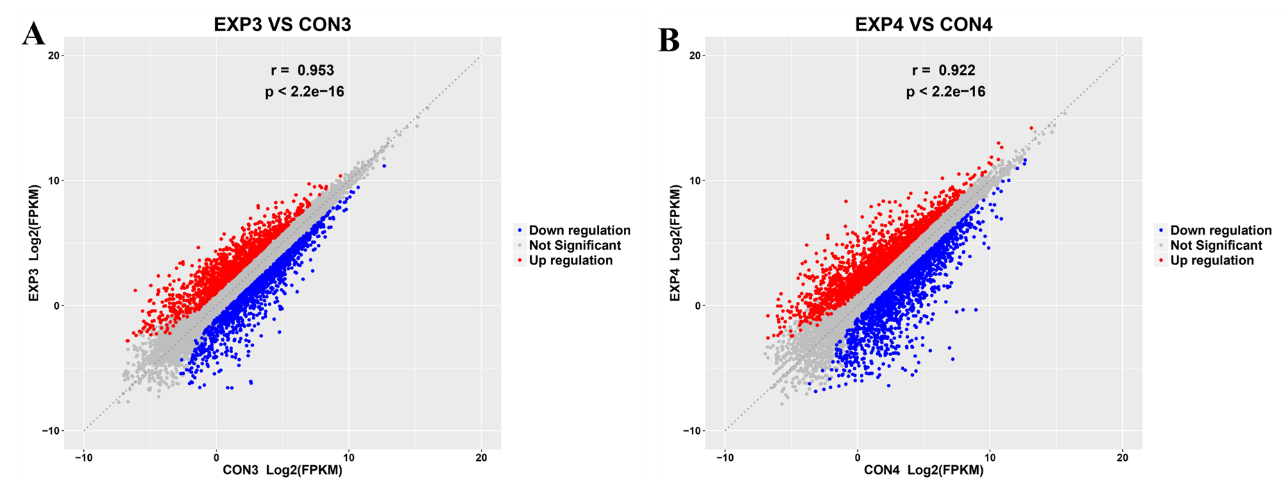
**

**Supplementary Figure 2.** DEGs between WQ1 and WQ2 at 10 and 18 DAP. A, WQ1 vs. WQ2 at 10 DAP; B, WQ1 vs. WQ2 at 18 DAP.


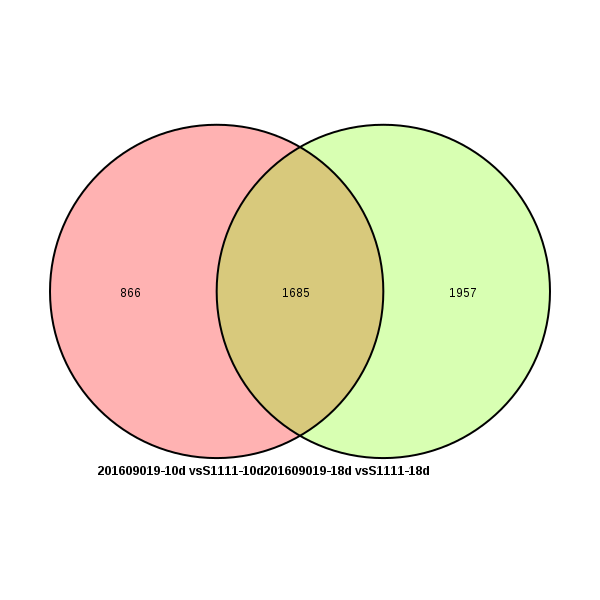


**Supplementary Figure 3.** DEGs between WQ1 and WQ2 at 10 and 18 DAP. Red round, WQ1 vs. WQ2 at 10 DAP; green round, WQ1 vs. WQ2 at 18 DAP.

**Supplementary Table 1.** The primers used for real-time PCR (qRT-PCR).

| Gene ID | Forward primer | Reverse primer |
| --- | --- | --- |
| *Cla97C02G043690* | GCCTCACGCCTCGCTATTGAAG | CTGAATGGAAGGGGAAGGGGTTTTC |
| *Cla97C02G043750* | GAGATTGTGATTGGAGGAGGCGATG | CCCTCTCCCTCTTCCTGCTCAAG |
| *Cla97C02G043800* | TTCTACGACGTTCAAGACCGATTGG | GCAGGAACAGAGGAACAAGCATTTG |
| *Cla97C02G043850* | GCCACTCCTCCTCCTGCCTATG | ACCAGTGCTTGTTCCAAAGAGACC |
| *Cla97C02G044050* | GCGAACGAGGGGTTTGTAAGAGG | GCTATTGCTCTGGCTATGGCTGTAG |
| *Cla97C02G044100* | CTCTGATACTTGGGAGGGCAAAGAC | CACCGTGGTACATCTTCTGGGATTG |
| *Cla97C02G044440* | TGTCTCCGATATGACCGTCCCATC | GCTAGGCACTTGTGGTTGATTGTTG |
| *Cla97C02G044520* | TTCTCCTCCTCCAGCAGCTTACTC | AATGGAGCTGGTTGTTTGTGGTTTG |
| *Cla97C05G094190*  *(GAPDH)* | GGGTGACATTCCGACCTACG | AGCTAGGCAGTTGGTAGTGC |

**Supplementary Table 2** Histological structure changes of pericarp development in WQ1 and WQ2.

| DAP | Material | Epidermal cell length (μm) | Epidermal cell thickness (μm) | Epidermal thickness (μm) | Exocarp cell area (μm^2^) | Mesocarp cell area (μm^2^) |
| --- | --- | --- | --- | --- | --- | --- |
| 10 | WQ1 | 29.19 ± 2.94^**^ | 18.61 ± 1.11 | 76.29 ± 6.99 | 529.40 ± 87.21^**^ | 2238.93 ± 610.78^**^ |
|  | WQ2 | 20.35 ± 2.29 | 18.53 ± 1.86 | 119.01 ± 6.83^**^ | 301.47 ± 100.92 | 808.40 ± 120.08 |
| 18 | WQ1 | 38.22 ± 3.46^**^ | 18.10 ± 1.37 | 78.17 ± 7.99 | 1047.6 ± 138.99^**^ | 3925.00 ± 676.08^**^ |
|  | WQ2 | 24.19 ± 0.97 | 19.97 ± 0.70* | 137.69 ± 10.60^**^ | 329.45 ± 48.49 | 942.19 ± 118.25 |

Note: Asterisks mark significant differences between WQ1 and WQ2 in the same stage according to Student’s *t*-test; ^**^*P*-value ≤ 0.01.

**Supplementary Table 3.** The coefficients of association among four fruit cracking related traits

|  | CTC | DFC | RH | RT |
| --- | --- | --- | --- | --- |
| CTC | 1.000 |  |  |  |
| DFC | 0.878^**^ | 1.000 |  |  |
| RH | 0.826^**^ | 0.655^**^ | 1.000 |  |
| RT | 0.660^**^ | 0.483^**^ | 0.544^**^ | 1.000 |

Note: Asterisks mark significant differences according to Student’s *t*-test; ^**^*P*-value ≤ 0.01.

**Supplementary Table 4.** SNP information of the parents and RILs.

| Sample | WQ1 | WQ2 | Offspring | Total |
| --- | --- | --- | --- | --- |
| SNP Num | 197,639 | 198,988 | 76,857.66 | 494,293 |

**Supplementary Table 5.** Flitted SNP information.

| Type | Num |
| --- | --- |
| Total marker | 494,293 |
| Parent marker lack | 218,708 |
| Depth not meet | 5,813 |
| No poly marker | 94,710 |
| Remain marker | 175,062 |

**Supplementary Table 6.** The genetic map information.

| LG ID | Marker Num | Total Distance | Average Distance  between marker | Gaps ≤ 5 | Max Gap |
| --- | --- | --- | --- | --- | --- |
| Cla97Chr01 | 373 | 132.98 | 0.36 | 99.19% | 9.09 |
| Cla97Chr02 | 179 | 129.76 | 0.73 | 98.31% | 10.51 |
| Cla97Chr03 | 357 | 127.57 | 0.36 | 98.88% | 18.58 |
| Cla97Chr04 | 113 | 63.48 | 0.57 | 96.43% | 9.74 |
| Cla97Chr05 | 538 | 139.67 | 0.26 | 99.26% | 6.24 |
| Cla97Chr06 | 317 | 104.59 | 0.33 | 98.42% | 15.9 |
| Cla97Chr07 | 269 | 117.5 | 0.44 | 97.76% | 10.88 |
| Cla97Chr08 | 241 | 121.27 | 0.51 | 97.92% | 11.91 |
| Cla97Chr09 | 330 | 144.02 | 0.44 | 97.87% | 8.66 |
| Cla97Chr10 | 313 | 121.81 | 0.39 | 98.08% | 8.23 |
| Cla97Chr11 | 305 | 120.09 | 0.4 | 98.36% | 8.63 |
| Total | 3,335 | 1,322.74 | 0.4 | 98.23% | 18.58 |

**Supplementary Table 7.** Evaluative results of samples sequencing data.

| Sample | Clean reads | Total mapped | Unique match | Q20 | GC content |
| --- | --- | --- | --- | --- | --- |
| 10d-1-1 | 57616037 | 85.18% | 84.00% | 97.17% | 43.00% |
| 10d-1-2 | 76320157 | 84.73% | 83.51% | 96.77% | 43.00% |
| 10d-1-3 | 68369077 | 82.60% | 81.20% | 97.38% | 43.00% |
| 10d-2-1 | 72366835 | 87.17% | 86.13% | 97.22% | 43.00% |
| 10d-2-2 | 75520002 | 85.96% | 84.73% | 97.29% | 42.00% |
| 10d-2-3 | 67758095 | 84.19% | 82.69% | 96.95% | 42.00% |
| 18d-3-1 | 62478792 | 80.17% | 78.64% | 97.13% | 42.00% |
| 18d-3-2 | 62501375 | 83.72% | 82.61% | 97.16% | 43.00% |
| 18d-3-3 | 62855682 | 81.12% | 79.87% | 97.40% | 43.00% |
| 18d-4-1 | 63776908 | 86.04% | 85.00% | 97.26% | 43.00% |
| 18d-4-2 | 68313788 | 82.67% | 81.21% | 97.08% | 42.00% |
| 18d-4-3 | 68915212 | 84.24% | 83.17% | 97.26% | 44.00% |
